# Prognostic significance of increased preoperative red cell distribution width (RDW) and changes in RDW for colorectal cancer

**DOI:** 10.1002/cam4.6036

**Published:** 2023-05-04

**Authors:** Xian Lu, Xiaofan Huang, Meng Xue, Zhenyu Zhong, Ran Wang, Wen Zhang, Lili Wang, Yuhan Qiao, Fei Ling, Qian Zhang, Yueying Zhang

**Affiliations:** ^1^ Jiangsu Province Key Laboratory of Anesthesiology Xuzhou Medical University Xuzhou Jiangsu China; ^2^ Department of Anesthesiology The Affiliated Hospital of Xuzhou Medical University Xuzhou Jiangsu China

**Keywords:** all‐cause mortality, changes in RDW, colorectal cancer, prognosis, RDW

## Abstract

**Background:**

Increased preoperative red cell distribution width (RDW) is associated with poor prognosis in several cancers, but the relationships between preoperative RDW and changes in RDW (ΔRDW) and colorectal cancer (CRC) prognosis remain unclear. Our study aimed to demonstrate the prognostic significance of increased preoperative RDW and ΔRDW for CRC.

**Methods:**

In this retrospective analysis, we enrolled 833 patients who underwent CRC surgery between 2015 and 2019 at the Affiliated Hospital of Xuzhou Medical University, China. ΔRDW in our study was defined as RDW at 1 month after discharge minus preoperative RDW. According to receiver operating characteristic (ROC) curve analysis, we used cut‐off values of 13.5% for RDW, 0.9% for ΔRDW. The cumulative survival rate was determined using the Kaplan–Meier method, and significant differences were evaluated by the log‐rank test. Multivariable Cox regression model was applied to clarify the independent risk factors for overall survival (OS), which were used to construct a nomogram prediction model. The competing risk method was also applied, and we analyzed only patients with early‐stage disease (stage 0‐II) for sensitivity analysis.

**Results:**

Multivariable Cox regression analysis demonstrated that age, RDW, ΔRDW, postoperative adjuvant chemotherapy, CEA, CA19‐9, ASA, TNM stage, and pathological type were independent factors for OS in CRC patients (all *p* < 0.05). These prognostic factors were used to establish and verify the OS nomogram. Poorer OS was linked to higher RDW (HR = 1.52; 95% CI, 1.11–2.08; *p* < 0.01) and ΔRDW (HR = 1.65; 95% CI, 1.19–2.28; *p* < 0.01) in all‐stage patients, and was only linked to higher RDW in early‐stage patients. In competing risk model, H‐RDW and H‐ΔRDW were confirmed to be independent risk factors for CSS in CRC patients.

**Conclusions:**

High preoperative RDW and ΔRDW are both risk factors for OS and CSS in CRC.

**Lay summary**

This study used information from CRC patients in the Affiliated Hospital of Xuzhou Medical University, China. We found that patients with increased preoperative RDW and ΔRDW tended to have a worse prognosis. Preoperative RDW combined with ΔRDW, postoperative adjuvant chemotherapy, age, ASA, TNM stage, histological type, CEA, and CA19‐9 can be used to accurately predict the prognosis of CRC patients. The results remind us that we need to concentrate on patients with high RDW in future work.

## 1 INTRODUCTION

Colorectal cancer (CRC) is the most common malignant tumor of the digestive tract and the fourth major cause of cancer‐related deaths globally, with 900,000 deaths annually.^1^ At present, the TNM stage is usually used to evaluate the prognosis of CRC. However, the TNM stage is based on the pathological results of surgically resected tumors, and the results are often delayed, so it cannot promptly predict the prognosis of CRC patients. After a patient's tumor is surgically removed, there is no dynamic indicator to predict the long‐term prognosis. However, the reduction in tumor markers can reflect the prognosis of patients to some extent. A considerable number of patients do not have high preoperative tumor markers, and these markers do not change much after surgery in some patients. Thus, a more accurate marker is needed to predict the prognosis of patients with CRC.

The red cell distribution width (RDW) is a metric used to describe the heterogeneity in the size of circulating red blood cells in a complete blood cell count. It is computed by dividing the mean red cell volume (MCV) by the standard deviation of the red cell volume (SD), and then multiplying that result by 100%. RDW was initially used to diagnose and differentiate anemia in combination with MCV.^2^ RDW was first described as an independent predictor of mortality in patients with chronic heart failure in 2007.^3^ Since then, RDW has been continuously confirmed to be associated with the diagnosis and prognosis of various diseases, such as cardiovascular diseases,^4^ respiratory diseases,^5^ liver diseases,^6^ kidney diseases,^7^ diabetes,^8^ thromboembolism,^9^ and cancer.^10^

In recent years, RDW has been studied as a potential prognostic factor for CRC. However, the prognostic role of RDW remains unclear, as published studies have shown different results. Some reports have confirmed that high preoperative RDW is a strong predictor of the diagnosis and poor prognosis of CRC,^11^, ^12^, ^13^ but some studies have also confirmed that high preoperative RDW is not an independent risk factor for poor prognosis in CRC patients.^14^ In addition, RDW was usually measured at a single time point (mainly at admission) in previous studies. There have been few relevant studies on the predictive value of changes in RDW for different diseases.^15^ Thus, the effect of dynamic changes in RDW in patients with CRC remains unknown. If we confirmed that changes in RDW could be used to predict prognosis, future clinical work could be focused on reducing RDW as an intervention to improve the prognosis of patients. After all, the value of RDW are relatively easy to be changed compared to TNM stage in cancer patients.

In our study, we retrospectively enrolled 833 patients who underwent surgery for CRC between 2015 and 2019 at the Affiliated Hospital of Xuzhou Medical University. This study aimed to assess the prognostic roles of preoperative RDW and changes in RDW in CRC patients.

## 2 METHODS

### 2.1 Ethics and registration

The Affiliated Hospital of Xuzhou Medical University's Research Ethics Committee (Xuzhou city, Jiangsu Province, China) approved this single‐center retrospective cohort study on March 1 (XYFY2022‐KL038‐01). The study was documented in the Chinese Clinical Trial Registry (ChiCTR2200056323). Individual consent was waived because we retrospectively collected information of patients in our study.

### 2.2 Study population

All patients undergoing primary tumor removal surgery for CRC between 2015 and 2019 at the Affiliated Hospital of Xuzhou Medical University Hospital in China were recruited. The inclusion criteria were as follows: patients with colorectal cancer who have been pathologically confirmed, aged 18 years or older. Exclusion criteria were: patients who experienced a second operation for a recurrence of CRC cancer, patients complicated with other tumors, perioperative blood transfusion and transfusion history within 3 months, patients who lacked perioperative RDW value prior to the operation and other clinical data.

### 2.3 Group setting

First, the preoperative RDW cut‐off value was considered based on the receiver operating characteristic (ROC) curve with 5‐year OS as the outcome. The RDW cohort was divided into H‐RDW and L‐ RDW using RDW = 13.5% as the cut‐off value(AUC = 0.56, *p* < 0.05).

Second, ΔRDW was defined as RDW at 1 month after discharge minus preoperative RDW. Similarly, the ΔRDW cohort was divided into H‐ΔRDW and L‐ΔRDW using ΔRDW = 0.9% as the cut‐off value according to ROC(AUC = 0.59, *p* < 0.05). (Figure S3).

### 2.4 Covariates

The baseline variables we collected included age, sex, history of preoperative chemoradiotherapy, body mass index (BMI), white blood cell (WBC) count, platelet count, serum albumin level, neutrophil count (NEUT), aspartate aminotransferase (AST), direct bilirubin, perioperative RDW, RDW at 1 month after discharge, serum CEA level, serum CA19‐9 level, ASA, the type of operation, TNM stage, histological type, histologic grade, tumor location, tumor size, and postoperative adjuvant chemotherapy. We also collected the information of comorbidities including hypertension, diabetes mellitus, coronary heart disease (CHD), chronic obstructive pulmonary disease (COPD), and history of stroke. The 8th Edition of the American Joint Committee on Cancer (AJCC) criteria were followed while reporting pathology specimens.

### 2.5 Follow up and outcomes

We used telephone interviews or electronic medical records to follow up the patient's survival until October 2022. The time of survival was calculated from the date of surgery to the most recent follow‐up or death.

The primary outcome of the study was overall survival (OS). OS was defined from the moment the patient received surgery until death, regardless of the cause. The secondly outcome was cancer‐specific survival (CSS). CSS was defined as the time from surgery to cancer‐related death.

### 2.6 Statistical analysis

The Kolmogorov–Smirnov test was performed to determine if the continuous data followed a normal distribution. Normally distributed quantitative data are expressed as the mean ± standard deviation (SD), and nonnormally distributed data are represented by the median and interquartile range (IQR). Categorical data are displayed as numbers and percentages. The Reverse Kaplan–Meier method was used to determine the median follow‐up period. The cumulative overall survival rate of CRC patients was determined using the Kaplan–Meier method, and significant differences were evaluated using the log‐rank test. Univariable Cox regression, all‐subset regression, and lasso regression were used to screen the initial variables. Then, these initial variables were separately put into the multivariable Cox regression for final screening the prognostic factors of OS. We used the backward stepdown process and selected final variables with *p* < 0.05. The final variables screened by the above methods were used to construct models predicting OS. The model with the largest area under the curve (AUC) was selected for constructing the nomogram. The effectiveness of RDW for OS prediction was evaluated using a receiver operating characteristic (ROC) curve. The calibration curve and concordance index (C index) were also employed to evaluate the model's performance. A bootstrap approach was applied to decrease the overfit bias. Deaths from colorectal cancer and deaths from other causes were treated as competing events in our study. To explore the possible effect of competing events on the risk factors of CRC patients, we performed cause‐specific hazards model to investigate the CSS of patients.

Sensitivity analysis was performed. In order to explore the role of preoperative RDW and changes in RDW in early‐stage CRC patients (stage 0‐II), we only analyzed the early‐stage CRC patients. Univariable and multivariable Cox regression analyses were performed to identify the risk factors of OS in early‐stage patients.

All statistics were performed with R, version 4.2.1. In addition, the Survival, Survminer, Ggplot2, Rms, Plyr, Forestplot, and Table One packages in R were used for statistical analysis.

## 3 RESULTS

### 3.1 Patient characteristics

Patients with relapse (*n* = 3), complications with other tumors (*n* = 2), perioperative blood transfusion and transfusion history within 3 months (*n* = 63), and incomplete records of clinical data (*n* = 13) were excluded. During the study, 865 patients met the inclusion criteria and were enrolled. A total of 833 patients were eventually followed up, while a total of 32 patients were lost to follow‐up. The follow‐up period ranged from 0.8 to 90.9 months, with a median of 60.1 months (95% CI, 59.1–61.1). At the end of the follow‐up, 176 patients had passed away. The 1, 3, and 5‐year OS rates were 97.3%, 86%, and 77.5%, respectively. The CSS rates at 1 year, 3 years, and 5 years were 97.6%, 86.5%, and 78.3%, respectively. Table 1 displays the baseline parameters and survival of patients.

**TABLE 1:** Baseline characteristics and survival situation.

Characteristics	*n*	Overall survival (%)		
		1 year	3 year	5 year
Sex				
Male	522 (62.7%)	97.7	87.2	76.1
Female	311 (37.3%)	96.5	83.9	79.7
Age (years)				
≤65	540 (64.8%)	98.9	88.4	82.1
>65	293 (35.2%)	94.3	81.5	68.9
BMI^a^				
<18.5 kg*m^2‐1^	42 (5.3%)	92.9	72.8	—
18.5–23.5 kg*m^2‐1^	364 (46.2%)	96.9	86.3	77
>23.5 kg*m^2‐1^	381 (48.5%)	97.9	87.2	78.9
Preoperative radiochemotherapy				
No	811 (97.4%)	97.3	86.2	77.6
Yes	22 (2.6%)	—	77.1	64.2
HTN				
No	573 (68.8%)	97.4	86.3	78.6
Yes	260 (31.2%)	96.9	85.3	74.9
DM				
No	720 (86.4%)	97.8	86.9	79.1
Yes	113 (13.6%)	94	80.4	—
CHD				
No	779 (93.5%)	97.3	86.1	77.2
Yes	54 (6.5%)	97.3	85.9	79
COPD				
No	799 (95.9%)	97.4	86.4	77.8
Yes	34 (4.1%)	95.1	75.8	—
History of stroke				
No	746 (89.5%)	97.4	86.3	78.7
Yes	87 (10.5%)	95.4	84.3	66.4
Anemia				
No	687 (82.5%)	97.4	86.6	78.4
Yes	146 (17.5%)	96.6	84	73.6
Albumin				
<40 g*L^‐1^	238 (28.6%)	94.9	81.5	71.9
≥40 g*L^‐1^	595 (71.4%)	98.1	87.8	79.6
AST				
Normal	787 (94.5%)	97.4	86.2	78.3
Abnormal	46 (5.5%)	94.5	82.8	60.9
DBIL^b^				
0–6 μmol*L^‐1^	730 (90.2%)	97.9	86.5	78.3
>6 μmol*L^‐1^	79 (9.8%)	92.1	82.9	73.1
ASA				
2	705 (84.6%)	98	88.4	79.2
3	126 (15.1%)	94.8	73.6	67.9
4	2 (0.3%)	—	—	—
The type of operation				
Laparoscopic surgery	731 (87.8%)	98.1	88	79.9
Open surgery	102 (12.2%)	91.3	71.3	60.3
TNM stage				
Stage 0–I	169 (20.3%)	99.2	97.6	94.6
Stage II	336 (40.3%)	98	92	84.2
Stage III	295 (35.4%)	95.6	79.3	65.9
Stage IV	33 (4%)	92.8	27	—
Histological type				
Adenocarcinoma	775 (93%)	97.3	87.1	78.5
Mucinous	45 (5.4%)	97.7	80.7	75.7
Others	13 (1.6%)	84.7	37.2	25.8
Histologic grade				
Well	66 (7.9%)	98	97	89.8
Moderate	551 (66.1%)	97.3	86.9	77.9
Poorly	170 (20.5%)	95.8	79.4	70.2
Unknown	46 (5.5%)	97.6	80.4	72.4
Location				
Colon	355 (42.6%)	95.5	84.1	76
Rectum	478 (57.3%)	98.5	87.4	78.5
Tumor size				
≤5 cm	597 (71.7%)	97.3	86.6	78.2
>5 cm	236 (28.3%)	97	84.3	75.9
CEA				
≤5 ng*mL^‐1^	450 (54%)	98.9	92.8	87.1
>5 ng*mL^‐1^	290 (34.8%)	96.7	79.6	67
Unknown	93 (11.2%)	90.5	73.2	62
CA19‐9				
≤37 U*mL^‐1^	644 (77.3%)	98.6	90	82.4
>37 U*mL^‐1^	89 (10.7%)	93.6	70.2	57.4
Unknown	100 (12.0%)	91.1	73.7	62.9
Postoperative adjuvant chemotherapy				
No	285 (34.2%)	94.4	80.1	72.3
Yes	548 (65.8%)	98.6	89.1	80.1
RDW				
<13.5%	580 (69.6%)	97.6	88.3	80.6
≥13.5%	253 (30.4%)	96.4	80.7	70.2
ΔRDW				
<0.9%	615 (73.8%)	97.9	89.6	82.1
≥0.9%	218 (26.2%)	95.4	76.2	64.3
RDW	13 (12.6–13.6)	97.3	86	77.5
ΔRDW	0.3 (−0.2–0.9)			
White blood cells (10^9^*L^‐1^)	6.2 (5.3–7.6)			
Neutrophil (10^9^*L^‐1^)	3.8 (3.0–4.9)			
Platelets (10^9^*L^‐1^)	236 (199.3–283)			
MCV	89.4 (86.1–92.2)			
MPV	10.5 (9.9–11.1)			

*Note*: Continuous data are presented as median (IQR).

Abbreviations: —, Survival rates are not shown because of the small cases; ASA, American society of anesthesiologists; CHD, coronary heart disease; COPD, chronic obstructive pulmonary disease; DM, diabetes mellitus; HTN, hypertension; MCV, mean corpuscle volume; MPV, mean platelet volume; RDW, red cell distribution width.

^a^ 46 patients were missing.

^b^ 24 patients were missing.

### 3.2 Survival analysis

**FIGURE 1:**
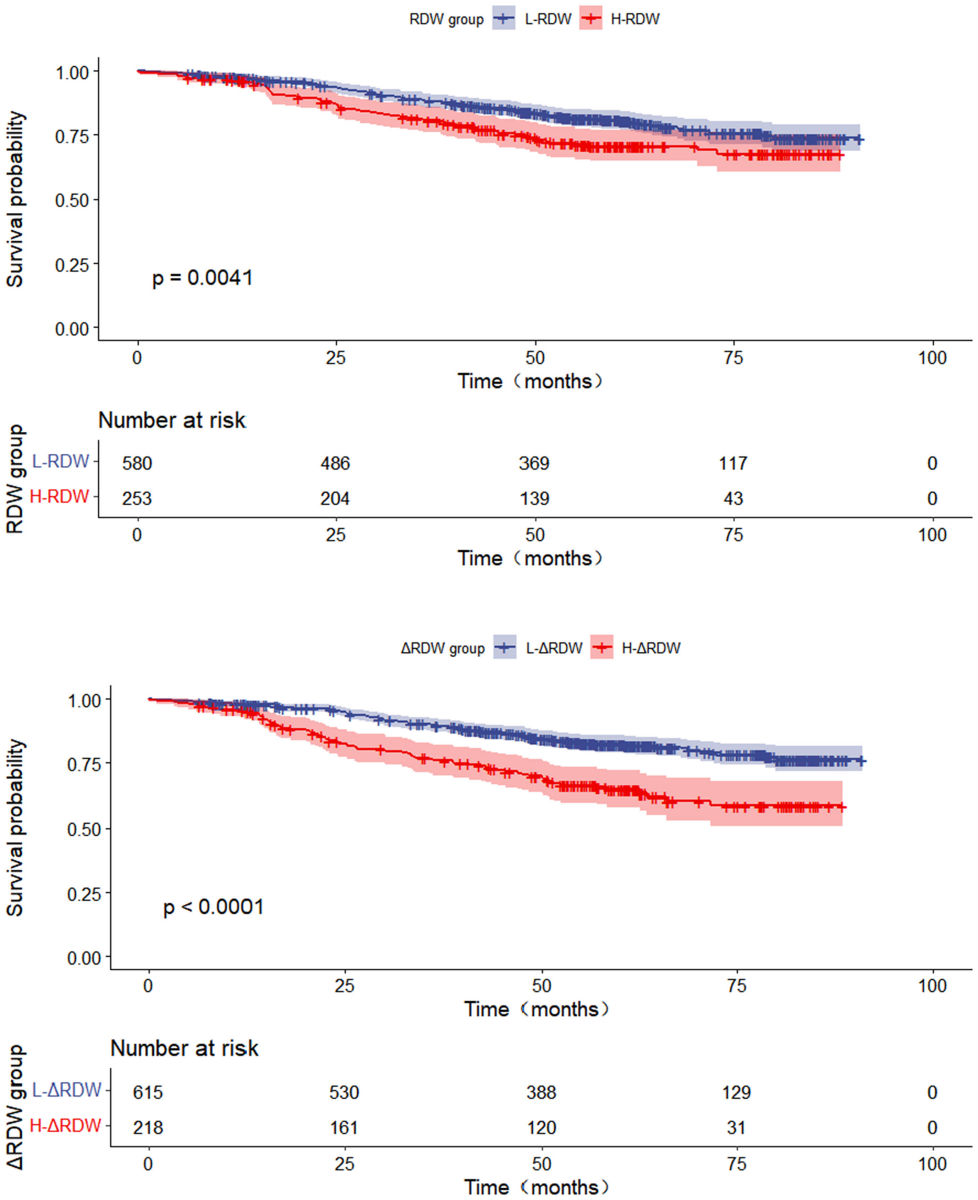
(A) Overall survival stratified by RDW (*p* = 0.004). (B) Overall survival stratified by ΔRDW (*p* < 0.001).

The Kaplan–Meier analysis revealed that the patients with increased RDW had a lower OS than those with RDW <13.5% (log‐rank *p* < 0.01; Figure 1A). Patients with ΔRDW ≥0.9% had a significantly higher overall mortality rate than those with ΔRDW <0.9% (log‐rank *p* < 0.01; Figure 1B). Similarly, the H‐RDW group showed lower CSS (log‐rank *p* < 0.01), and the H‐ΔRDW group also had worse CSS (log‐rank *p* < 0.01). (We did not show the figure of Kaplan–Meier analysis of CSS stratified by RDW and ΔRDW).

### 3.3 Cox regression analysis and construction of the prediction model

**FIGURE 2:**
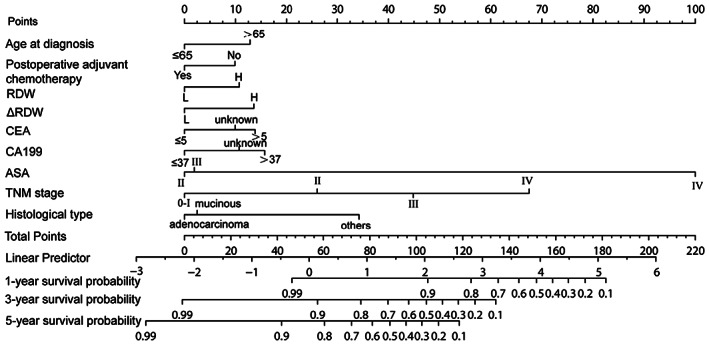
Nomogram prediction model for predicting OS in patients with colorectal cancer.

We found that the variables screened by univariable Cox regression had the best AUC of the model, so the variables were selected for multivariable Cox regression. Univariable Cox analysis showed that age, history of stroke, RDW, lower albumin, AST, CEA, CA19‐9, ΔRDW, ASA, the type of operation, TNM stage, pathological type, differentiation, postoperative adjuvant chemotherapy and neutrophil count were prognostic factors of CRC patients (all *p* < 0.05, Table 2). In multivariable analysis, RDW was proven to be an independent prognostic factor of OS in CRC (hazard ratio [HR] = 1.52; 95% confidence interval [CI], 1.11–2.08; *p* < 0.01), and ΔRDW was also a prognostic value in CRC (hazard ratio [HR] = 1.65; 95% confidence interval [CI], 1.19–2.28).These independent risk factors (RDW, ΔRDW, age, ASA, TNM stage, histological type, postoperative adjuvant chemotherapy, CEA and CA19‐9) were selected to establish an OS risk prediction model for CRC patients (Figure 2). We applied the bootstrap validation method to validate our model internally. The area under the curve (AUC) of the receiver operating characteristic (ROC) curve of 1, 3, 5‐year OS were 0.86 (95% CI, 0.79‐0.92), 0.84 (95% CI, 0.80‐0.88) and 0.81 (95% CI, 0.77–0.86) (Figure S1). The calibration curve of the nomogram was close to the ideal reference line, indicating that the prediction of OS was consistent with the actual OS (Figure S2).

**TABLE 2:** Univariate and multivariate analysis in prognosis of colorectal cancer patients.

Characteristics	Univariate analysis HR (95% CI)	*p* value	Multivariate analysis HR (95% CI)	*p* value
Sex		0.42	—	—
Male	1.14 (0.83–1.55)			
Female				
Age (years)		<0.01		<0.01
≤65				
>65	1.93 (1.44–2.59)		1.71 (1.19–2.45)	
BMI^a^		0.22	—	—
<18.5 kg*m^2‐1^				
18.5–23.5*kg m^2‐1^	0.66 (0.36–1.20)			
>23.5 kg*m^2‐1^	0.09 (0.32–1.71)			
History of preoperative radiochemotheray		0.48	—	—
No				
Yes	1.34 (0.59–3.02)			
HTN		0.3	—	—
No				
Yes	1.18 (0.87–1.61)			
DM		0.25	—	—
No				
Yes	1.26 (0.85–1.88)			
CHD		0.63	—	—
No				
Yes	0.86 (0.47–1.59)			
COPD		0.55	—	—
No				
Yes	1.24 (0.61–2.52)			
History of stroke		0.03		0.89
No				
Yes	1.57 (1.03–2.38)		0.97 (0.62–1.51)	
Anemia		0.39		
No				
Yes	1.18 (0.81–1.70)			
Albumin		0.019		0.47
<40 g*L^‐1^				
≥40 g*L^‐1^	0.69 (0.51–0.94)		1.14 (0.80–1.61)	
WBC (10^9^*L^‐1^)	1.05 (0.98–1.12)	0.17	—	—
Neu (10^9^*L^‐1^)	1.09 (1.02–1.17)	0.02	1.01 (0.94–1.08)	0.81
PLT (10^9^*L^‐1^)	1 (1–1)	0.71	—	—
AST		0.02		0.06
Normal				
Abnormal	1.89 (1.13–3.15)		1.73 (0.97–3.06)	
DBIL^b^		0.27	—	—
0–6 μmol*L^‐1^				
>6 μmol*L^‐1^	1.31 (0.83–2.08)			
ASA		<0.01		<0.01
2				
3	1.75 (1.22–2.52)	0.03	1.10 (0.74–1.65)	
4	165.33 (33.1–825.83)	<0.01	39.55 (7.24–216.15)	
The type of operation		<0.01		0.37
Laparoscopic surgery				
Open surgery	2.23 (1.56–3.19)		1.21 (0.80–1.84)	
TNM stage		<0.01		<0.01
Stage 0–I				
Stage II	3.37 (1.60–7.10)	0.01	2.75 (1.27–5.93)	
Stage III	8.03 (3.89–16.54)	<0.01	5.96 (2.81–12.62)	
Stage IV	33.81 (15.24–75)	<0.01	14.10 (6.04–32.90)	
Histological type		<0.01		<0.01
Adenocarcinoma				
Mucinous	1.07 (0.548–2.103)	0.84	1.74 (0.76–3.99)	
Others	5.66 (3.07–10.45)	<0.01	5.54 (2.40–12.78)	
Histologic grade		0.01		0.16
Well				
Moderate	3.01 (1.23–7.37)	0.02	1.73 (0.69–4.31)	
Poorly	4.37 (1.73–11.01)	<0.01	1.96 (0.76–5.05)	
Unknown	3.86 (1.36–10.96)	0.01	0.8 (0.23–2.79)	
Location		0.62	—	—
Colon				
Rectum	0.86 (0.64–1.16)			
Tumor size		0.89	—	—
≤5 cm				
>5 cm	1.02 (0.74–1.42)			
CEA		<0.01		<0.01
≤5 ng*mL^‐1^				
>5 ng*mL^‐1^	2.78 (1.99–3.89)	<0.01	1.70 (1.18–2.43)	
Unknown	3.32 (2.14–5.16)	<0.01	1.42 (0.26–7.76)	
CA19‐9		<0.01		0.01
≤37 U*mL^‐1^				
>37 U*mL^‐1^	2.84 (1.94–4.15)	<0.01	1.94 (1.29–2.91)	
Unknown	2.32 (1.56–3.44)	<0.01	1.46 (0.27–7.86)	
Postoperative adjuvant chemotherapy		<0.01		0.02
No				
Yes	0.67 (0.49–0.9)		0.65 (0.45–0.93)	
RDW		<0.01		0.02
<13.5%				
≥13.5%	1.56 (1.15–2.11)		1.52 (1.11–2.08)	
ΔRDW		<0.01		<0.01
<0.9%				
≥0.9%	2.24 (1.66–3.02)		1.65 (1.19–2.28)	

^a^ 46 patients were missing.

^b^ 24 patients were missing.

### 3.4 Competing‐risk models

Deaths from CRC and other causes were considered competing events in our study. After adjusting for age, ASA, TNM stage, pathological type, postoperative adjuvant chemotherapy, CEA and CA19‐9, H‐RDW was confirmed to be an independent risk factor for CSS in CRC patients (HR = 1.54; 95% CI, 1.12–2.24; *p* < 0.01, Table 3), and H‐ΔRDW was associated with a lower CSS (HR = 1.78; 95% CI, 1.29–2.44; *p* < 0.01, Table 3).

**TABLE 3:** Sensitivity analysis.

	Competing‐risk models		Sensitivity analysis	
	HR (95% CI)	*p*	HR (95% CI)	*p*
RDW				
<13.5%		<0.01		0.01
≥13.5%	1.54 (1.12–2.24)		2.01 (1.18–3.42)	
ΔRDW				
<0.9%		<0.01		0.24
≥0.9%	1.78 (1.29–2.44)		1.44 (0.79–2.63)	

*Note*: Competing‐risk models, Hazard ratios (HRs) and 95% confidence intervals (95% CIs) of RDW and ΔRDW were calculated by cause‐specific hazards model; Sensitivity analysis, Hazard ratios (HRs) and 95% confidence intervals (95% CIs) were calculated by Cox proportional hazards models when inclued early‐stage patients (stage 0–II).

### 3.5 Sensitivity analysis

We analyzed only 505 early‐stage patients (stage 0‐II), and RDW was divided into H‐RDW and L‐ RDW using RDW = 13.5% as the cut‐off value (AUC was 0.67, *p* < 0.01). Univariable Cox analysis showed that age, patients with diabetes mellitus, history of stroke, RDW, AST, CEA, CA19‐9, ΔRDW, ASA, the type of operation, TNM stage and neutrophil count were prognostic factors of CRC patients (all *p* < 0.05). In multivariable analysis, we found that patients with elevated preoperative RDW tended to have a worse survival rate (HR = 2.01; 95% CI, 1.18–3.42; *p* = 0.01) (Table 3); however, high ΔRDW group did not show a worse OS than low ΔRDW group.

## 4 DISCUSSION

In this retrospective study, we enrolled patients of all disease stages and found that high preoperative RDW and ΔRDW were risk factors for the prognosis of patients with CRC; furthermore, this relationship persisted after sensitivity analysis. In addition, we established and validated a novel nomogram to improve predictive accuracy based on RDW and ΔRDW. RDW was more predictive of OS in early‐stage patients than in all‐stage patients, but ΔRDW was not a prognostic factor for early‐stage patients. To the best of our knowledge, this is the first investigation to explore the influence of changes in RDW on the prognosis of patients with CRC and to build a nomogram of CRC based on RDW.

A growing number of studies in recent years have revealed that RDW is a prognostic factor for many diseases, such as cardiovascular disease, respiratory disease, liver disease, kidney disease, diabetes, thromboembolism, and cancers.^4^, ^5^, ^6^, ^7^, ^8^, ^9^, ^10^ However, previous studies only analyzed the prognostic role of preoperative RDW (a single quantitative indicator) and did not consider the role of RDW changes in the prognosis of patients. Thus, there have been some subsequent efforts to investigate the role of changes in RDW in patients. High ΔRDW was discovered to be a predictor of early adverse complications after coronary artery bypass grafting in the study of Lee et al^16^ However, the role of ΔRDW in CRC patients is still unclear. Our study was the first to investigate the effect of ΔRDW on prognosis in CRC patients.

Previous studies have investigated the association between increased RDW and overall mortality in CRC patients. However, the study population and conclusions were not consistent. Zhang et al^17^ retrospectively enrolled 625 rectal cancer patients who underwent radical resection and found that high RDW was linked to a lower disease‐free survival (DFS). In a retrospective study of 168 CRC patients, Li et al^12^ discovered that higher RDW was associated with lower 3‐year and 5‐year DFS and OS. Pedrazzani et al^14^ analyzed 591 CRC patients to explore whether RDW is a prognostic factor for OS and CSS. In multivariate analysis, they observed that preoperative RDW was not an independent predictor of OS or DFS, but the H‐RDW group had a lower 10‐year OS. They also found that the association between preoperative RDW and OS only existed in stage I CRC patients.

However, none of the above studies took into account comorbidities such as hypertension, diabetes, coronary heart disease, stroke history, and nutritional status. These potential factors significantly affect the survival of patients and are nonnegligible confounding factors. Therefore, Cheng et al^18^ used the propensity score matching method (PSM)to balance out these confounding factors. They retrospectively enrolled 5315 patients with stage I or II disease who underwent radical surgery. After PSM, it was found that the higher the RDW was, the worse the OS, DFS and CSS were. Although confounding factors can be adjusted for by PSM, this method is not perfect. PSM will cause a loss of sample size, and if the number of lost cases is too large, selection bias caused by matching cannot be ruled out. Most of the above studies did not exclude patients with perioperative blood transfusion and a recent history of blood transfusion. To the best of our knowledge, blood transfusion can significantly increase the value of RDW,^19^ interfering with the judgment of the prognostic value of RDW.^20^ Patients with CRC are more prone to experience chronic blood loss than those with other malignancies, and perioperative blood transfusion is more common in CRC patients than in those with other diseases.^21^ If we did not exclude the population with perioperative blood transfusion, the value of RDW would fluctuate significantly and thus interfere with the accuracy of the findings.

Therefore, the strengths of our study were as follows. First, we considered the effect of confounding factors such as a history of blood transfusion and comorbidities. Second, we investigated whether changes in RDW were an independent prognostic factor in CRC patients. Third, we conducted sensitivity analyses to eliminate the impact of different stages in CRC patients, as well as competing risk models to capture the potential contribution of several competing events. Fourth, we established a predictive nomogram of OS in CRC based on RDW.

We finally concluded that (1) there was an association between increased preoperative RDW and OS and CSS in all‐stage CRC patients, which was consistent with the findings of previous studies.^14^, ^18^ Moreover, we found that RDW had a more significant risk effect of OS in the early‐stage CRC patients. (2) ΔRDW was an independent risk factor for OS and CSS in CRC patients after multivariate regression. Moreover, compared to single preoperative RDW, ΔRDW have an increased prognostic value for predicting OS in CRC patients. However, high ΔRDW did not show a worse OS in early‐stage CRC patients. Previous studies have shown that the change in RDW over time have additive prognostic value compared to single preoperative RDW. Xiao et al^22^ found that high ΔRDW was associated with cardiovascular disease outcomes when the interval of two RDW measurements was 4 months. Oh et al^23^ concluded that measurement of RDW at 1 month after acute decompensated heart failure (ADHF) assisted in the prediction of adverse cardiovascular (CV) outcomes. (3) The results of multivariate analysis showed that age, RDW, ΔRDW, postoperative adjuvant chemotherapy, CEA, CA19‐9, ASA, TNM stage and pathological type were independent factors forOS in CRC patients. These risk factors were used to establish and verify the nomogram for predicting 1‐year, 3‐year, and 5‐year OS. The survival rate of CRC patients in different age groups varied greatly. Patients older than 65 years had a worse OS, consistent with the results of other studies.^24^ CEA and CA19‐9 are recognized as prognostic indicators in patients with CRC,^25^ as is the case with ASA and TNM stage.^26^ Our study found that compared with adenocarcinoma and mucinous adenocarcinoma, patients with other pathological types had a worse prognosis, which was related to the fact that the other types of adenocarcinomas in our study were mainly signet‐ring cell carcinoma.

The mechanism of the association between elevated RDW and poor prognosis in CRC patients remains unclear. It is speculated that the following three mechanisms may be involved: (1) In the process of tumor development, cancer‐related inflammation plays a critical role.^27^ The release of proinflammatory cytokines could lead to the inhibition of the activity of erythropoietin. So, under an inflammatory state, red blood cells would tend to be produced ineffectively and be damaged easily, leading to increased RDW levels.^18^, ^28^, ^29^ RDW was proven to be associated with IL‐6, TNF‐α, hepcidin and other cytokines that can affect the biological behavior of tumor cells in previous study.^30^, ^31^ Therefore, inflammation may contribute to the association between increased RDW levels and decreased survival. (2) Oxidative stress can make red blood cells fragile and accelerate their maturity and lifespan, resulting in increased RDW.^32^ Therefore, greater oxidative stress may play a role in the relationship between higher RDW and worse survival. (3) Malignant tumors tend to be wasting diseases, and patients with cancer often have poor nutrition. The insufficient intake of nutrients and micronutrients, especially vitamin B12, folic acid, iron and other hematopoietic raw materials, suppresses erythropoiesis and alters the deformability of the erythrocyte membrane, resulting in increased RDW and ΔRDW.

The current study has several limitations. First, this was a single‐center retrospective study with a biased population selection, so further prospective studies are needed to demonstrate the role of RDW and ΔRDW in CRC patients. Second, statistically speaking, RDW was an independent prognostic risk factor in all‐stage CRC patients, but HR of RDW was 1.52, which was not very large, indicating that this single factor did not seem to play a very significant role in theOS of the colorectal cancer. There's no denying that RDW combined with ΔRDW, postoperative adjuvant chemotherapy, age, ASA, TNM stage, histological type, CEA, and CA19‐9 can accurately predict the OS of CRC patients. However, in the sensitivity analysis of early‐stage patients, we found that the prognostic value of RDW in OS was more significant than that of all‐stage patients. It can be explained that the TNM stage plays an increasingly important role in the prognosis of patients with advanced cancer. In our sensitivity analysis, we may not have assessed enough population to explore the role of RDW in early‐stage patients. Third, RDW = 13.5% and ΔRDW = 0.9% were defined as the cut‐off values according to the ROC curves of 5‐year OS in this study, which were only applicable to our study population. Further multicenter large sample studies are needed to explore appropriate cut‐off values. In conclusion, we found that preoperative RDW (≥13.5%) and ΔRDW (≥0.9%) were independent predictors of OS and CSS in CRC patients. Future clinical work should pay attention to CRC patients with elevated RDW and adopt a scientific intervention strategy to improve the prognosis of CRC patients.

## AUTHOR CONTRIBUTIONS

**Xian Lu:** Conceptualization (equal); data curation (equal); formal analysis (equal); investigation (equal); methodology (equal); writing – original draft (equal); writing – review and editing (equal). **Xiaofan Huang:** Conceptualization (equal); data curation (equal); formal analysis (equal); methodology (equal); writing – review and editing (equal). **Meng Xue:** Conceptualization (equal); investigation (equal); methodology (equal); writing – review and editing (equal). **Zhenyu Zhong:** Conceptualization (equal); methodology (equal); writing – review and editing (equal). **Ran Wang:** Conceptualization (equal); methodology (equal); writing – review and editing (equal). **Wen Zhang:** Conceptualization (equal); methodology (equal); writing – review and editing (equal). **Lili Wang:** Conceptualization (equal); investigation (equal); methodology (equal). **Yuhan Qiao:** Conceptualization (equal); investigation (equal); methodology (equal). **Fei Ling:** Conceptualization (equal); investigation (equal); methodology (equal). **Qian Zhang:** Conceptualization (equal); investigation (equal); methodology (equal). **Yueying Zhang:** Conceptualization (equal); methodology (equal); writing – original draft (equal); writing – review and editing (equal).

## FUNDING INFORMATION

This research did not receive any specific grant from funding agencies in the public, commercial, or not‐for‐profit sectors.

## CONFLICT OF INTEREST STATEMENT

The authors declare that they have no conflicts of interest.

## Supporting information


Figure S1

Figure S2

Figure S3

## Data Availability

Research data are not shared.
