# Supplementary material for: Prognostic significance of increased preoperative red cell distribution width (RDW) and changes in RDW for colorectal cancer
Source: Cancer Med. 2023 May 4;12(12):13361–73. doi: 10.1002/cam4.6036 (PMC10315724; doi:10.1002/cam4.6036)
Supplement: Supplementary file 1 — Figure S1 [file CAM4-12-13361-s002.doc]

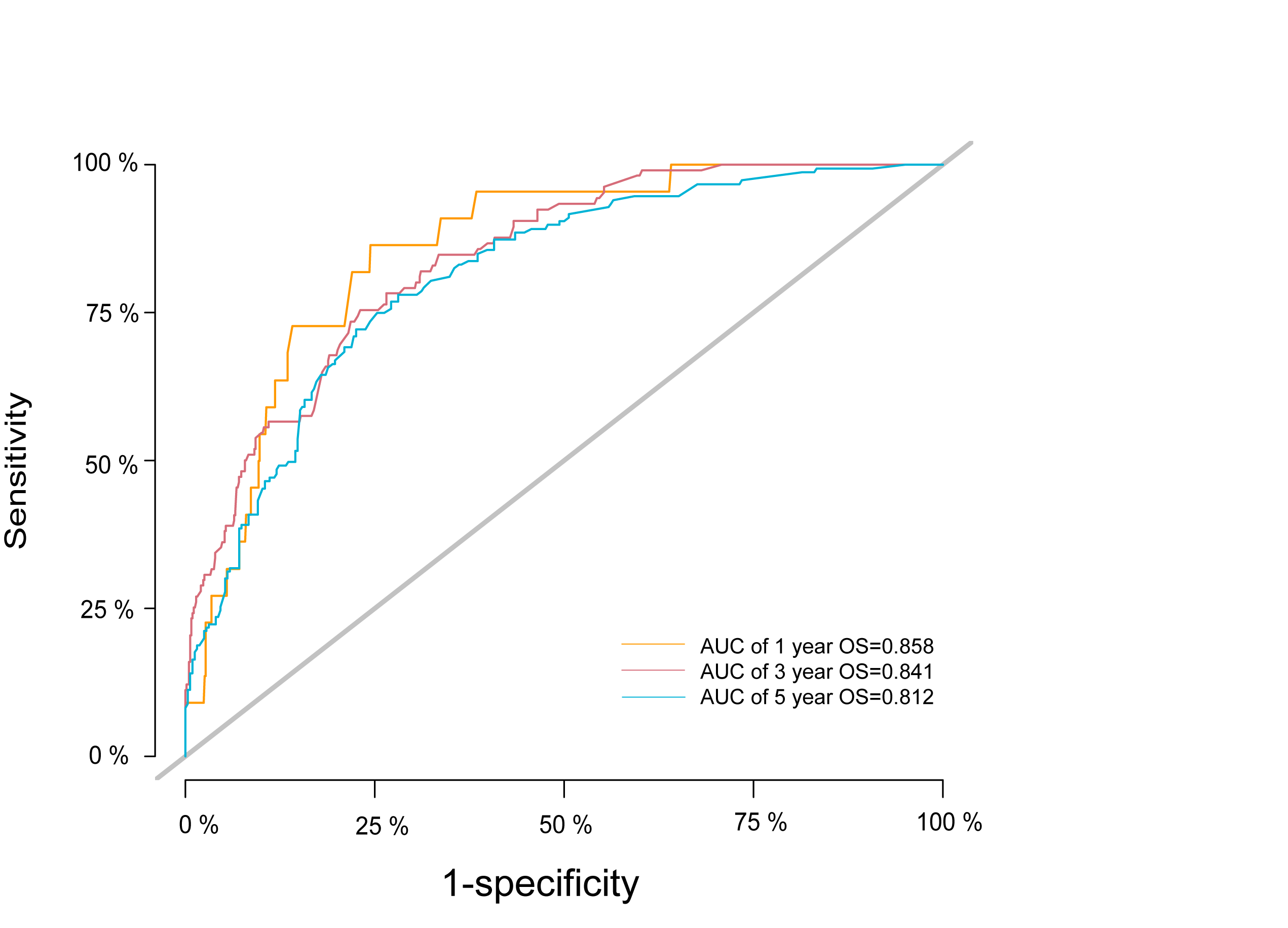


Figure 3 The area under the curve (AUC) of the receiver operating characteristic (ROC) curve for predicting 1-year,3-year and 5-year OS.
