# Supplementary material for: Prognostic significance of increased preoperative red cell distribution width (RDW) and changes in RDW for colorectal cancer
Source: Cancer Med. 2023 May 4;12(12):13361–73. doi: 10.1002/cam4.6036 (PMC10315724; doi:10.1002/cam4.6036)
Supplement: Supplementary file 2 — Figure S2 [file CAM4-12-13361-s001.doc]

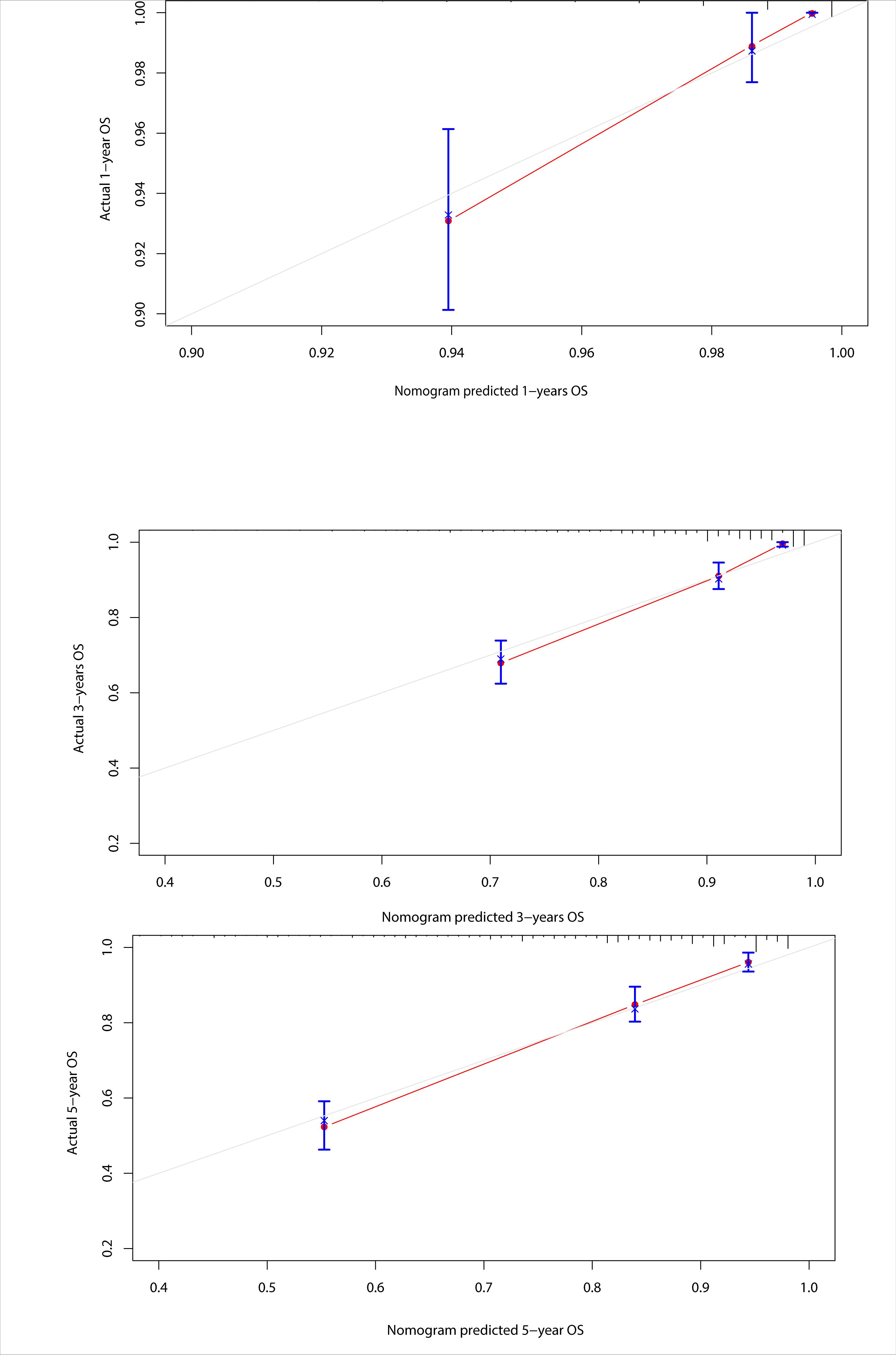


Supplementary Fig 2 calibration curve for1-year, 3-year, 5-year overall survival.Dashed line indicates ideal reference line where predicted probabilities would match the observed proportions.The red line is the calibrated optimal probability. Dashes represent nomogram-predicted probabilities grouped for each of the three tripartite groups.
