# Supplementary material for: Prognostic significance of increased preoperative red cell distribution width (RDW) and changes in RDW for colorectal cancer
Source: Cancer Med. 2023 May 4;12(12):13361–73. doi: 10.1002/cam4.6036 (PMC10315724; doi:10.1002/cam4.6036)
Supplement: Supplementary file 3 — Figure S3 [file CAM4-12-13361-s003.doc]

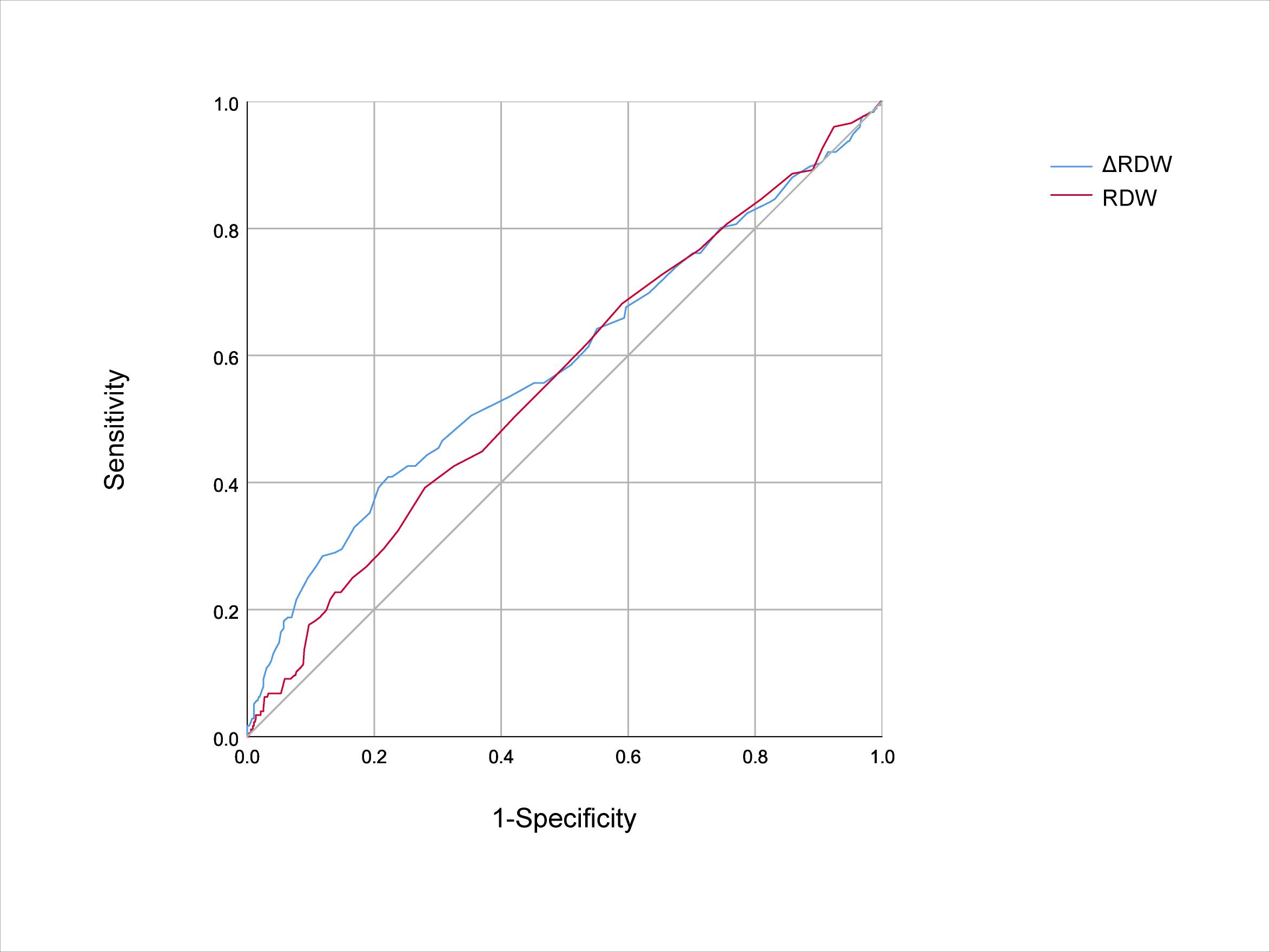


Supplementary Fig 3. ROC analysis of RDW, ΔRDW and 5-year overall survival. Area under curve (AUC) was 0.56, 0.59. Sensitivity and specificity were 39% and 72%, 42% and 78%.
